# Draft Genome Sequence of *Salmonella enterica* subsp. *enterica* Serovar Lille Strain CRJJGF_000101 (Phylum *Gammaproteobacteria*)

**DOI:** 10.1128/genomeA.00603-16

**Published:** 2016-07-14

**Authors:** Sushim K. Gupta, Elizabeth A. McMillan, Charlene R. Jackson, Prerak T. Desai, Steffen Porwollik, Michael McClelland, Lari M. Hiott, Shaheen B. Humayoun, Jonathan G. Frye

**Affiliations:** a Bacterial Epidemiology and Antimicrobial Resistance Research Unit, U.S. National Poultry Research Center, U.S. Department of Agriculture, Agricultural Research Service, Athens, Georgia, USA; b Department of Microbiology, University of Georgia, Athens, Georgia, USA; c Department of Microbiology and Molecular Genetics, University of California Irvine, Irvine, California, USA

## Abstract

Here, we report a 4.98 Mbp draft genome sequence of *Salmonella enterica* subsp. *enterica* serovar Lille strain CRJJGF_000101, isolated from ground beef in 2007.

## GENOME ANNOUNCEMENT

Salmonellosis is a major public-health concern worldwide. Consumption of raw or improperly prepared or contaminated foods, mostly of animal origin, is considered the major route of human salmonellosis (1). There are more than 2,500 serovars of Salmonella, exhibiting more than 46 different O groups and 114 different H groups; all of them are considered a potential threat to human health (2). The *Salmonella* strain CRJJGF_000101 was isolated from ground beef in 2007 using standard microbiology techniques and serotyped using SMART (3). The isolate was serogrouped using serogroup-specific antisera (Difco Laboratories, Detroit, MI) and the serovar was determined at the National Veterinary Services Laboratories, APHIS, USDA (Ames, IA). This bacterium belonged to antigenic group O:7(C1), along with *Salmonella* serovar Rumford, and contained somatic O antigen 7 and only phase1 flagellar H antigens z38 (7:z38:-) (4). Using pulsed-field gel electrophoresis (PFGE) as described by PulseNet (5), the isolate was assigned PFGE pattern LPPX01.0020. MICs (µg/ml) were determined by broth microdilution using the Sensititre semi-automated antimicrobial susceptibility system (TREK Diagnostics Systems, Thermo Fisher Scientific, Inc., Oakwood Village, OH). Results were interpreted according to Clinical and Laboratory Standards Institute (CLSI) guidelines (6).

The genomic DNA was isolated using a GenElute bacterial genomic DNA kit (Sigma-Aldrich, St. Louis, MO) and the DNA library was constructed using a Nextera-XT DNA preparation kit and paired-end sequencing was performed on an Illumina HiSeq2500 (Illumina Inc., San Diego, CA) using a 500-cycle MiSeq reagent kit. About 4,304,692 reads with quality score >30 were assembled using Velvet assembler (7), which resulted in 196 contigs with minimum contig length ≥200 bp. The total assembly size was 4.98 Mbp, with *N*_50_ values of 70.7 kbp, and G+C content of 51.98%. The contigs were ordered with MAUVE using *Salmonella* LT2 as a reference (8), and prodigal (9) and ARAGORN (10) were used to predict coding sequences (CDS) and tRNAs. A total of 4,764 coding sequences (≥50 amino acids) and 48 tRNAs were predicted within the genome. Prophages, signal peptides, and resistance genes were predicted using PHAST (11), signalp (12), and ARG-ANNOT (13), respectively. We identified signal peptides in 462 genes, two clustered regularly interspaced short palindromic repeat (CRISPR) (14) loci, and 5-intact/6-incomplete phages in the genome. We detected an *aac6-Iaa* resistance gene, which remains cryptic and a tetracycline resistance gene *tetC*, which was confirmed phenotypically. The detected arsenic resistance genes (*pst*BACS) (15) correlated with the phenotypes. The strain’s MIC was 411 and 58 µg/ml compared to a wild type median MIC, of 51 and 15 µg/ml for arsenate and arsenite, respectively. The pathogenesis analysis of *Salmonella* serovar Lille in the mouse model categorized the strain in the low invasive group (16). However, *Salmonella* serovar Lille has been reported during salmonellosis outbreaks (17). Host specific invasion may be the reason for low invasiveness in mice. The information generated from the genome may enhance our understanding of the variations in the virulence genes for host specific infection.

### Nucleotide sequence accession number.

The genome sequence of *Salmonella enterica* subsp. *enterica* serovar Lille strain CRJJGF_000101 has been deposited in GenBank under the accession number JQWF00000000. This paper describes the first version of the genome.
